# Robotic-assisted versus laparoscopic versus open liver resection: comparison of postoperative outcomes according to the IWATE difficulty score

**DOI:** 10.1007/s00464-025-12231-0

**Published:** 2025-09-17

**Authors:** Schaima Abdelhadi, Mohamad El-Ahmar, Sepehr Abbasi Dezfouli, Katharina Vedder, Maike Hermann, Vanessa Orth, Mahmoud Halawa, Meik Moennichs, Christoph Reissfelder, Flavius Sandra-Petrescu

**Affiliations:** 1 Department of Surgery, Medical Faculty Mannheim, Universitätsmedizin Mannheim, Heidelberg University, Mannheim, Germany; 2 DKFZ Hector Cancer Institute at the University Medical Center, Mannheim, Germany

**Keywords:** Minimally invasive liver surgery, Robotic liver resection, Laparoscopic liver surgery, IWATE score, Hepatectomy, Surgical outcomes

## Abstract

**Background:**

Minimally invasive liver surgery (MILS) has become increasingly established, yet the relative benefits of laparoscopic (LLR) and robotic-assisted liver resection (RLR) compared with open liver resection (OLR) across different levels of surgical difficulty remain debated. This study aimed to compare perioperative outcomes of RLR, LLR, and OLR stratified by the IWATE difficulty score.

**Methods:**

All consecutive patients undergoing elective liver resection between April 2018 and December 2024 at a high-volume hepatobiliary center were retrospectively analyzed from a prospectively maintained database. Patients were stratified into low/intermediate (IWATE 0–6) and advanced/expert (IWATE 7–12) groups. Multivariable regression and interaction term analyses were performed to adjust for confounders and assess the modifying effect of surgical difficulty.

**Results:**

A total of 686 patients were included: 425 (62%) underwent LLR, 101 (15%) RLR, and 160 (23%) OLR. Of these, 400 (58%) were advanced/expert resections. Minimally invasive approaches were associated with significantly reduced blood loss, morbidity, and length of stay compared with OLR across all IWATE levels. In advanced/expert resections, RLR provided the greatest benefit, with lower major complications (8% vs. 17% LLR vs. 23% OLR) and shorter length of stay (median 6 vs. 9 days OLR). Multivariable analyses confirmed these findings, with both LLR (OR 0.24, 95% CI 0.10–0.55) and RLR (OR 0.24, 95% CI 0.06–1.00) independently associated with reduced major complications compared to OLR. Interaction analyses demonstrated that the comparative advantage of RLR was most pronounced in advanced/expert resections, while LLR showed particular efficiency in low/intermediate cases.

**Conclusion:**

Both LLR and RLR are safe and effective across all levels of surgical difficulty. RLR, however, offers distinct advantages in technically demanding advanced and expert cases. These findings reinforce the role of MILS as the preferred standard and highlight the importance of tailoring the surgical approach to case complexity.

**Supplementary Information:**

The online version contains supplementary material available at 10.1007/s00464-025-12231-0.

Over the past decades, laparoscopic liver resection (LLR) has become an established and widely accepted technique in liver surgery [1]. Numerous studies have demonstrated that LLR offers several advantages over open liver resection (OLR), including reduced intraoperative blood loss, shorter hospital stays, and lower postoperative morbidity [2, 3]. Despite these benefits, LLR has inherent technical limitations. Restricted instrument mobility, the lack of depth perception due to two-dimensional imaging, and ergonomically demanding working conditions can pose challenges, particularly in more complex resections [4, 5].

In recent years, robotic surgical systems have gained increasing interest and recognition among surgeons. Robotic-assisted liver resection (RLR) offers several technical improvements over conventional laparoscopy. These include articulated instruments with enhanced degrees of freedom, stable high-definition three-dimensional visualization, and tremor filtration [6–8]. These features may help facilitate technically demanding resections that would otherwise be challenging with standard laparoscopy. Nevertheless, the clinical advantages of RLR remain controversial and are still a matter of ongoing debate [9–11]. While some studies have reported improved perioperative outcomes with RLR, particularly in complex resections, others have found comparable results to LLR with longer operative times and higher associated costs [6, 9–11].

When evaluating surgical outcomes, the technical difficulty of the procedure must be considered. The IWATE criteria provide a validated and widely used scoring system for assessing the complexity of liver resections based on tumor location, size, extent of resection, proximity to major vessels, and liver function [12].

Therefore, this study aimed to compare perioperative outcomes of RLR, LLR, and OLR stratified by IWATE difficulty levels based on a large single-center cohort treated at a high-volume hepatobiliary center.

## Methods

### Study design and patient cohort

All consecutive patients who underwent liver surgery between April 2018 and December 2024 were identified from a prospectively maintained institutional database at the Department of Surgery, University Hospital Mannheim, Heidelberg University [13–16]. Patients were eligible for inclusion if they were aged 18 years or older and underwent elective liver resection. Exclusion criteria were emergency liver resections, multivisceral resections, and procedures involving biliodigestive anastomosis. The latter were excluded as such procedures are more commonly performed in open surgery and are considered a major risk factor for postoperative morbidity [17]. This cohort study was conducted by the STROCSS guidelines and approved by the Heidelberg University ethics committee (2024-839) [18]. The study was retrospectively registered in the German Clinical Trials Register (DRKS00036636).

### Definitions and data acquisition

Liver resections were classified according to the Brisbane 2000 terminology [19]. Anatomic Liver resections were defined in line with Couinaud’s portal segmentation system as the complete removal of one or more portal territories and the corresponding hepatic parenchyma [19]. The IWATE score was used to assess the technical difficulty of liver resections [12]. Resections with an IWATE score of 0–6 were categorized as low/intermediate difficulty, while scores of 7–12 were classified as advanced/expert. Patients who required conversion from a laparoscopic or robotic approach to open surgery were analyzed according to the initial surgical approach, following the intention-to-treat principle.

We extracted demographic and preoperative clinical data, including comorbidities and laboratory parameters: age, sex, body mass index (BMI), American Society of Anesthesiologists (ASA) physical status classification, cardiovascular and pulmonary comorbidities, diabetes mellitus, presence of liver cirrhosis, Child–Pugh classification, underlying liver disease, prior treatments, and laboratory values such as albumin, bilirubin, international normalized ratio (INR), platelet count, alkaline phosphatase, gamma-glutamyltransferase (GGT), aspartate aminotransferase (AST), and alanine aminotransferase (ALT).

Additional intraoperative and postoperative parameters were collected, including the extent of resection, the use and duration of the Pringle maneuver, the use of intrahepatic inferior vena cava (IVC) clamping, operative time, intraoperative blood loss, histopathological findings, postoperative length of hospital stay, and postoperative complications. Histopathological data included tumor size, resection margin status, and the presence of microvascular invasion.

Postoperative complications were classified according to the Clavien–Dindo classification. Liver-specific complications were defined and reported in accordance with the criteria established by the International Study Group of Liver Surgery (ISGLS); only complications of grade B and C were considered [20, 21].

The primary endpoint was the occurrence of postoperative complications within 90 days after surgery.

### Standardization of perioperative care

All patients underwent standardized pre-, intra-, and postoperative care according to institutional protocols within a structured framework. In oncologic cases, surgical indications were discussed preoperatively during multidisciplinary team meetings. All procedures were performed by experienced attending hepatobiliary surgeons with expertise in open, laparoscopic, and robotic liver surgery.

### Operative technique

OLR was performed with the patient in the supine position using a reversed L-shaped incision, as previously described [13–15, 22]. In all cases, intraoperative hepatic ultrasound (IOUS) was utilized before resection to assess resectability, detect vascular invasion, and determine the transection plane. Parenchymal transection was carried out under low central venous pressure (< 5 cm H₂O), using bipolar forceps and a clamp-crushing technique in combination with energy devices (LigaSure™, Medtronic, Minneapolis, MN, USA; Thunderbeat™, Olympus Medical Systems Corp., Tokyo, Japan). Intrahepatic vessels were divided using linear staplers, titanium clips, or Hem-o-lok clips. The Pringle maneuver was intermittently applied using an umbilical tape or a Foley catheter placed around the hepatoduodenal ligament.

In the minimally invasive groups, patients were placed in the reverse Trendelenburg position [13, 15, 16, 23] and a pneumoperitoneum of 12 mmHg was established. The detailed trocar placement for both LLR and RLR has been published previously [13, 15, 16, 23]. Parenchymal transection in the LLR group was performed using bipolar forceps and a crush-clamp technique in combination with advanced sealing devices (LigaSure™; Thunderbeat™). In the RLR group, parenchymal transection was carried out using either the scissor hepatectomy technique or a vessel sealer on the daVinci Xi or *X* platform (Intuitive Surgical, Sunnyvale, CA, USA). Specimens were extracted via a Pfannenstiel incision or by reopening previous abdominal scars. Intra-abdominal drains were not routinely placed.

### Statistical analysis

Statistical analyses were performed using Jamovi software, version 2.6.26. Categorical variables are presented as frequencies and percentages and were compared using the Pearson *χ*^2^ test or Fisher’s exact test, as appropriate. Continuous variables are expressed as mean ± standard deviation (SD) or median with interquartile range (IQR), depending on their distribution. Comparisons between groups were made using the two-tailed Student’s t-test for normally distributed data or the Mann–Whitney U test for non-normally distributed data. A *p*-value of < 0.05 was considered statistically significant.

To address potential confounding, multivariable regression analyses were performed for the main outcomes, major complications (Clavien–Dindo ≥ 3), and postoperative length of stay. Three prespecified model specifications were applied: Model A was fully adjusted for all baseline covariates and the IWATE score, Model B was a parsimonious model adjusted only for repeat hepatectomy and multiple resection sites, and Model C was a parsimonious model adjusted for age, sex, BMI, and ASA score. Results are reported as odds ratios (OR) with 95% confidence intervals (CI) for binary outcomes and regression coefficients (β) with 95% CI for continuous outcomes.

In addition, interaction term analyses were conducted to evaluate whether the effect of surgical approach on outcomes differed according to IWATE difficulty level. For binary outcomes, logistic regression models with interaction terms between surgical approach and IWATE score were used, while for continuous outcomes, linear regression models with corresponding interaction terms were applied. Estimated marginal means and predicted probabilities were calculated and presented with 95% CI.

## Results

**Fig. 1:**
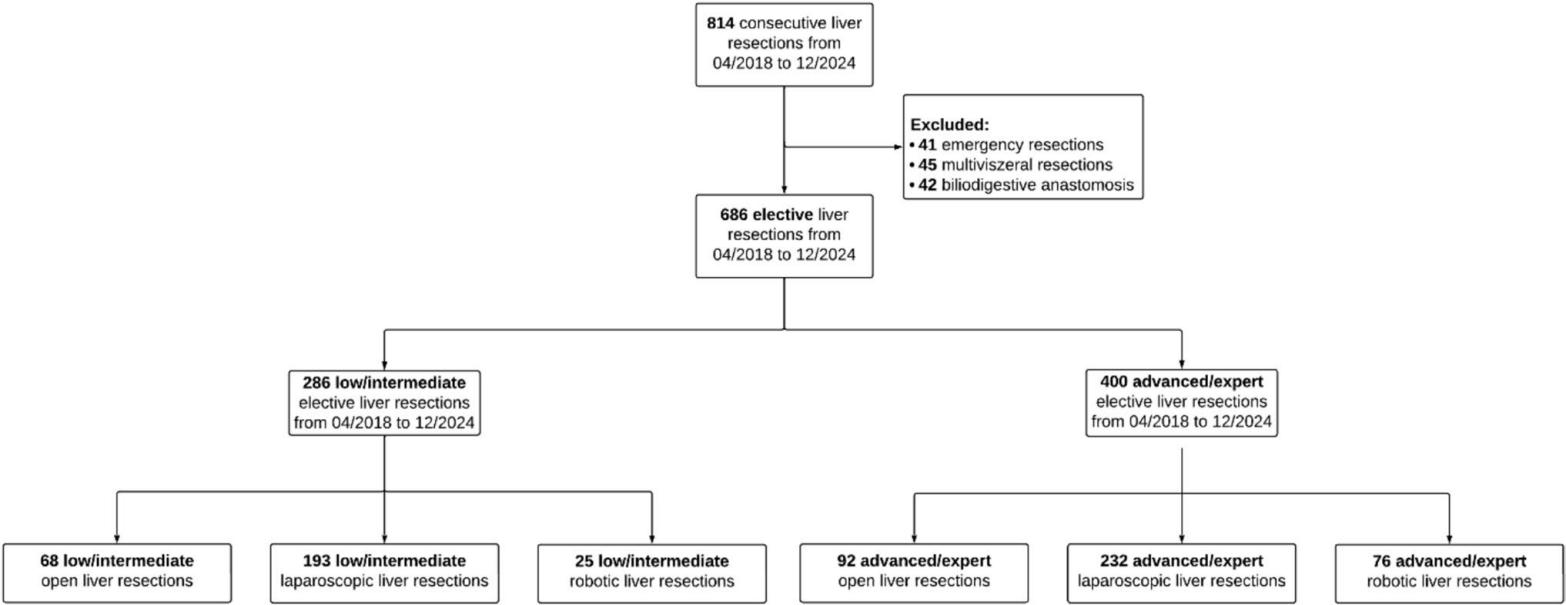
Patient flow chart

Eight hundred and fourteen consecutive liver resections were performed at our institution between April 2018 and December 2024. After excluding 41 emergency resections, 45 multivisceral resections, and 42 procedures involving biliodigestive anastomosis, 686 patients who underwent elective liver resections were included in the final analysis. The study cohort was subsequently stratified according to the IWATE difficulty score [12]. Based on this classification, 286 resections (42%) were categorized as low/intermediate difficulty and 400 resections (58%) as advanced/expert. Each group was further subdivided according to the surgical approach. Among the low/intermediate resections, 68 (24%) were performed via an open approach, 193 (67%) laparoscopically, and 25 (9%) using a robotic-assisted technique. In the advanced/expert group, 92 (23%) resections were performed via open surgery, 232 (58%) laparoscopically, and 76 (19%) using a robotic approach (Fig. 1).

### Patient characteristics

Patient demographic and clinical characteristics are summarized in Table 1. Baseline characteristics were well balanced between the groups, with no statistically significant differences regarding age, BMI, medical comorbidities, or the underlying diagnosis leading to liver resection. However, patients in the OLR group had a significantly higher rate of previous liver resections compared to those in the LLR and RLR groups (29 vs. 19 vs. 10%, *p* < 0.01).

**Table 1:** Baseline characteristics of the study cohort

Baseline characteristics of the study cohort	OLR (*n* = 160)	LLR (*n* = 425)	RLR (*n* = 101)	*p*-value
Age, years^a^	61 (52–69)	65 (56–74)	67 (56–77)	0.11
BMI, kg/m^2a^	26 (23–30)	26 (23–30)	25 (22–28)	0.91
Sex ratio, male: female	97:63	246:179	47:54	0.06
ASA				0.38
I	8 (5)	20 (4)	6 (6)	
II	82 (51)	215 (51)	41 (41)	
III	67 (42)	183 (43)	52 (51)	
IV	3 (2)	7 (2)	2 (2)	
Cardiovascular comorbidities	74 (46)	237 (56)	49 (49)	0.06
Diabetes mellitus	39 (24)	94 (22)	20 (20)	0.73
Pulmonary comorbidities	17 (11)	66 (16)	16 (16)	0.20
Liver cirrhosis	12 (8)	54 (13)	13 (13)	0.08
Child A	10 (6)	46 (11)	10 (10)	
Child B	2 (1)	8 (2)	3 (3)	
Etiology of cirrhosis				0.35
Alcohol	5 (3)	27 (6)	7 (7)	
Viral	6 (4)	17 (4)	5 (5)	
Hepatitis B	4 (3)	11 (3)	3 (3)	
Hepatitis C	2 (1)	6 (1)	2 (2)	
MASLD	1 (1)	10 (2)	1 (1)	
Previous treatment				
Previous abdominal surgery	58 (36)	123 (29)	26 (26)	***0.03***
Open	23 (40)	44 (36)	10 (38)	
Minimally invasive	35 (60)	79 (64)	16 (61)	
Previous liver resection	47 (29)	80 (19)	10 (10)	** < *0.01***
Open	19 (40)	28 (35)	4 (40)	
Minimally invasive	28 (60)	52 (65)	6 (60)	
Previous locoregional therapy	4 (3)	17 (4)	2 (2)	0.51
Previous systemic treatment	23 (14)	60 (14)	11 (11)	0.86
Diagnosis				0.06
PHB malignancy	49 (31)	135 (32)	32 (32)	
HCC	21 (13)	90 (21)	20 (20)	
CCC	25 (16)	42 (10)	11 (11)	
GBC	3 (2)	3 (1)	1 (1)	
Metastatic disease	83 (52)	188 (44)	42 (42)	
Benign	28 (17)	102 (24)	27 (26)	

Values presented in bold italics indicate statistically significant results (*p* < 0.05)

^a^Values are median (interquartile range)

^b^Values are mean (standard deviation)

*BMI* body mass index, *ASA* American Society of Anesthesiologists, *INR* international normalized ratio, *AP* alkaline phosphatase, *gGT* gamma glutamyltransferase, *AST* aspartate aminotransferase, *ALT* alanine aminotransferase, *PHB* primary hepatobiliary, *HCC* hepatocellular carcinoma, *CCC* cholangiocellular carcinoma, *GBC* gallbladder cancer

### Operative characteristics

A subgroup analysis was performed to further evaluate operative characteristics and perioperative outcomes according to the IWATE difficulty score. Operative characteristics of patients undergoing low/intermediate and advanced/expert liver resections are summarized in Tables 2 and 3.

**Table 2:** Operative characteristics and outcomes of advanced/expert hepatectomy

Operative characteristics and outcomes of advanced/expert hepatectomy	OLR (*n* = 92)	LLR (*n* = 232)	p-value (OLR vs LLR)	RLR (*n* = 76)	p-value (OLR vs RLR)	p-value (LLR vs. RLR)
Surgical procedure			0.07		0.06	0.09
Non-anatomic resections	26 (28)	70 (30)		20 (26)		
Right (extended) hepatectomy	35 (38)	56 (24)		17 (22)		
Left (extended) hepatectomy	16 (17)	27 (12)		7 (9)		
Left lateral sectionectomy	3 (3)	8 (3)		2 (3)		
Right posterior sectionectomy	3 (3)	14 (6)		4 (5)		
Right anterior sectionectomy	1 (1)	1 (1)		5 (7)		
Other mono- or bisegmentectomies	23 (25)	90 (39)		24 (31)		
Conversion	–	29 (12)		3 (4)		***0.03***
Operative time, min^a^	251 (182–351)	261 (194–355)	0.56	320 (246–404)	***0.005***	***0.005***
Pringle maneuver	56 (60)	136 (59)	0.18	60 (79)	0.83	0.19
Duration, min^a^	43 (26–58)	34 (19–62)	0.22	56 (33–92)	***0.05***	** < *0.001***
Blood loss, ml^a^	950 (500–1850)	650 (300–1400)	***0.01***	500 (288–1100)	***0.008***	***0.03***
Resection margins			0.97		0.96	0.99
R0	70 (95)	197 (96)		53 (96)		
R1	4 (5)	9 (4)		2 (4)		
Postoperative complications^b^	49 (53)	99 (42)	***0.03***	16 (21)	***0.001***	***0.03***
Grade I	17 (18)	37 (16)		4 (5)		
Grade II	11 (12)	27 (10)		5 (7)		
Grade III	12 (13)	20 (9)		3 (4)		
Grade IV	4 (4)	7 (3)		1 (1)		
Grade V	6 (6)	10 (4)		3 (4)		
Type of complications						
Wound infection	13 (14)	10 (4)	0.28	1 (1)	***0.01***	0.41
Burst abdomen	4 (4)	5 (2)	0.15	0 (0)	0.07	0.31
Pleural effusion with atelectasis	10 (11)	16 (7)	0.21	2 (3)	***0.04***	0.51
Pulmonary embolism	3 (3)	4 (2)	0.96	2 (3)	0.23	0.06
Posthepatectomy hemorrhage^c^	2 (2)	6 (3)	0.11	1 (1)	0.68	0.57
Posthepatectomy bile leakage^c^	7 (8)	18 (8)	0.99	4 (5)	0.81	0.54
Posthepatectomy liver failure^c^	5 (5)	13 (6)	0.98	2 (3)	0.96	0.34
Length of stay, d^a^	9 (6–18)	6 (5–10)	** < *0.001***	6 (4–9)	***0.002***	0.34

Values presented in bold italics indicate statistically significant results (*p* < 0.05)

^a^Values are median (interquartile range)

^b^Clavien-Dindo classification

^c^ISGLS- Classification; only grade B and C considered

*IVC* infrahepatic vena cava, *R0* no residual tumor, *R1* microscopic residual tumor

**Table 3:** Operative characteristics and outcomes of low/intermediate hepatectomy

Characteristics	OLR (*n* = 68)	LLR (*n* = 193)	p-value (OLR vs LLR)	RLR (*n* = 25)	p-value (OLR vs RLR)	p-value (LLR vs. RLR)
Surgical procedure			0.47		0.56	0.67
Non-anatomic resections	45 (66)	107 (55)		12 (48)		
Left lateral sectionectomy	10 (15)	45 (23)		6 (24)		
Other mono- or bisegmentectomies	13 (19)	41 (21)		7 (28)		
Conversion		13 (7)		1 (4)		0.82
Operative time, min^a^	164 (85–240)	119 (77–180)	***0.02***	180 (122–237)	0.47	***0.003***
Pringle maneuver	17 (25)	59 (30)	0.34	10 (40)	0.29	0.33
Duration, min^a^	19 (11–20)	24 (15–42)	0.13	23 (17–34)	0.12	0.33
Blood loss, ml^a^	300 (100–500)	100 (10–300)	***0.03***	150 (50–300)	***0.04***	0.48
Resection margins			0.97		0.98	0.96
R0	54 (95)	109 (96)		18 (95)		
R1	3 (5)	5 (4)		1 (5)		
Postoperative complications^b^	28 (41)	39 (20)	***0.04***	3 (12)	** < *0.001***	0.66
Grade I	14 (21)	14 (7)		1 (4)		
Grade II	6 (9)	6 (3)		1 (4)		
Grade III	3 (4)	13 (7)		0 (0)		
Grade IV	3 (4)	3 (1)		0 (0)		
Grade V	2 (3)	3 (1)		1 (4)		
Type of complications						
Wound infection	6 (9)	1 (1)	** < *0.01***	0 (0)	***0.05***	0.72
Burst abdomen	3 (4)	1 (1)	***0.02***	0 (0)	0.29	0.72
Pleural effusion with atelectasis	7 (10)	5 (2)	***0.01***	1 (4)	***0.04***	0.09
Pulmonary embolism	2 (3)	0 (0)	***0.02***	0 (0)	0.39	–
Posthepatectomy hemorrhage^c^	2 (3)	2 (1)	0.23	0 (0)	0.29	0.61
Posthepatectomy bile leakage^c^	3 (4)	7 (4)	0.81	0 (0)	0.29	0.33
Posthepatectomy liver failure^c^	2 (3)	0 (0)	0.11	0 (0)	0.39	–
Length of stay, d^a^	7 (5–10)	4 (3–6)	***0.002***	4 (4–5)	***0.006***	0.63

Values presented in bold italics indicate statistically significant results (*p* < 0.05)

^a^Values are median (interquartile range)

^b^Clavien-Dindo classification

^c^ISGLS- Classification; only grade B and C considered

*IVC* infrahepatic vena cava, *R0* no residual tumor, *R1* microscopic residual tumor

In the subgroup of advanced/expert liver resections, the distribution of the type of resection was comparable between the three surgical approaches. The conversion rate from a minimally invasive to an open approach was significantly higher in the LLR group compared to RLR (12 vs. 4%, *p* = 0.03). Both RLR and LLR were associated with significantly lower intraoperative blood loss compared to OLR (500 and 650 ml vs. 950 ml, *p* = 0.008 and *p* = 0.01, respectively). The median operative time was significantly longer in the RLR group than in OLR and LLR (320 vs. 251 min and 261 min, *p* = 0.005).

In the low/intermediate liver resections subgroup, non-anatomic resections were the most frequently performed procedures across all surgical approaches. Conversion rates from minimally invasive to open surgery were 7% in the LLR groups and 4% in the RLR group (*p* = 0.82). Intraoperative blood loss was significantly lower in LLR and RLR compared to OLR (100 and 150 ml vs. 300 ml; *p* = 0.03 and *p* = 0.04, respectively). Operative time was significantly longer in the RLR group compared to LLR (180 vs. 119 min, *p* = 0.003), whereas OLR procedures showed similar operative times to RLR (*p* = 0.47).

### Postoperative outcomes

Postoperative outcomes are presented in Tables 2 and 3. In the advanced/expert group, overall postoperative complication rates were significantly lower in the RLR group (21%) compared to LLR (42%, *p* = 0.03) and OLR (53%, *p* = 0.001). Major complications (Clavien-Dindo grade ≥ 3) occurred less frequently in the RLR group (8%) compared to LLR (17%) and OLR (23%). The length of hospital stay was significantly shorter in the RLR and LLR groups compared to OLR (median 6 vs. 9 days; *p* = 0.002 and *p* < 0.001, respectively).

In the low/intermediate group, the overall postoperative complication rate was also lowest in the RLR group (12%) compared to LLR (20%, *p* = 0.66) and OLR (41%, *p* < 0.001). Major complications (Clavien-Dindo grade ≥ 3) were observed in 4% of RLR cases, 7% of LLR cases, and 8% of OLR cases. The length of hospital stay was significantly shorter in the minimally invasive groups (RLR and LLR) than in OLR (median 4 vs. 7 days; *p* = 0.006 and *p* = 0.002, respectively).

### Multivariable and interaction analyses

In multivariable regression analyses, both laparoscopic and robotic hepatectomy remained independently associated with reduced major complications (Clavien-Dindo grade ≥ 3) compared to open resection. In the fully adjusted model (Model A), the odds ratio for major complications was 0.24 (95% CI 0.10–0.55, *p* < 0.001) for LLR vs. OLR and 0.24 (95% CI 0.06–1.00, *p* = 0.032) for RLR vs. OLR. These findings were confirmed in parsimonious sensitivity models (Model B and Model C), with LLR (OR 0.45, 95% CI 0.26–0.78, *p* = 0.004) and RLR (OR 0.38, 95% CI 0.13–1.11, *p* = 0.049) remaining favorable compared to OLR (Supplementary Table 1).

For postoperative length of stay, both LLR and RLR were associated with significantly shorter hospitalization than OLR across all models. In the fully adjusted model (Model A), the reduction was –11.8 days for LLR (95% CI –16.2 to –7.4, *p* < 0.001) and –13.9 days for RLR (95% CI –21.2 to –6.3, *p* < 0.001) compared to OLR. Similar results were observed in the parsimonious models (Model B: –7.8 and –9.1 days; Model C: –8.0 and –8.1 days for LLR and RLR vs. OLR, all *p* < 0.01; (Supplementary Table 2).

**Fig. 2:**
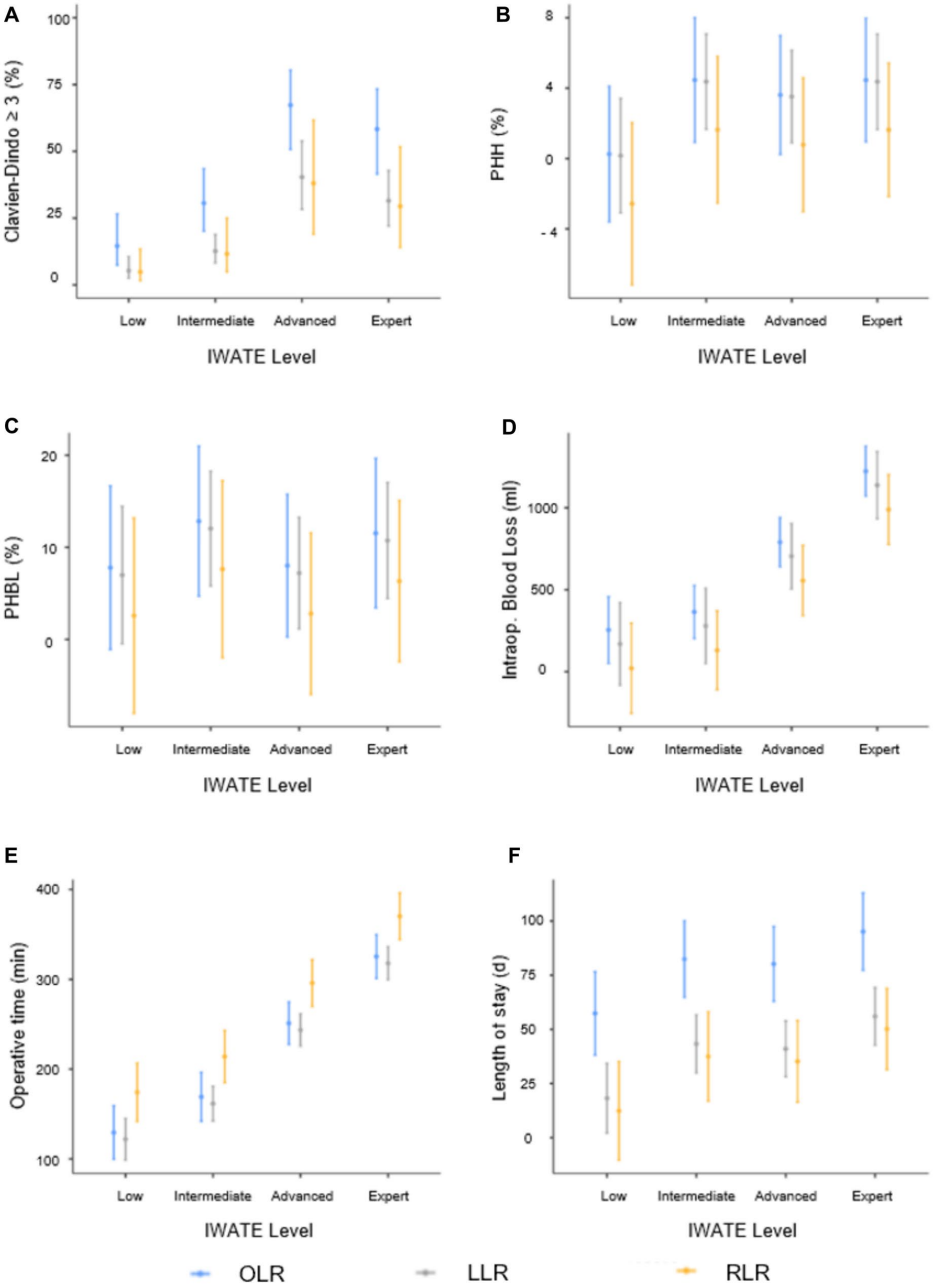
Interaction analysis of surgical approach and IWATE difficulty level for perioperative outcomes. Shown are predicted probabilities or means (± 95% CI) for **A** major morbidity (Clavien–Dindo ≥ III), **B** posthepatectomy hemorrhage (PHH), **C** posthepatectomy bile leakage (PHBL), **D** intraoperative blood loss, **E** operative time, and **F** length of stay (LOS), stratified by IWATE level and surgical approach (blue = Open Liver Resection (OLR), grey = Laparoscopic Liver Resection (LLR), yellow = Robotic Liver Resection (RLR))

In an interaction term analysis stratified by IWATE difficulty levels, perioperative outcomes consistently favored minimally invasive approaches over OLR (Fig. 2). The reduction in major complications (Clavien-Dindo grade ≥ 3) and postoperative hospital stay after LLR and RLR was most pronounced in advanced and expert resections. For major complications (Clavien-Dindo grade ≥ 3), the probability increased stepwise with IWATE level in all groups but remained consistently lower after minimally invasive approaches. In advanced resections, the estimated probability of major complications (Clavien-Dindo grade ≥ 3) was 67% (95% CI 51–80) for OLR, compared to 40% (95% CI 28–54) for LLR and 38% (95% CI 19–62) for RLR. Postoperative hospital stay likewise increased with IWATE level, but was significantly shorter after minimally invasive surgery. For expert resections, the mean LOS was 19.0 days (95% CI 15.5–22.6) after OLR, compared to 11.2 days (95% CI 8.5–13.9) for LLR and 10.0 days (95% CI 6.3–13.8) for RLR. Similarly, intraoperative blood loss was significantly lower in both minimally invasive groups across all IWATE levels. Operative time was longest in RLR, particularly in advanced and expert resections, whereas rates of bile leakage and posthepatectomy hemorrhage showed no significant differences between approaches.

## Discussion

In this large single-center cohort study, we compared perioperative outcomes of RLR, LLR, and OLR stratified by IWATE difficulty levels [12]. Our results demonstrate that both minimally invasive approaches (RLR and LLR) outperform OLR in terms of reduced intraoperative blood loss, shorter hospital stays, and lower postoperative morbidity across all levels of surgical difficulty [1–6]. These advantages were particularly pronounced in advanced/expert resections, where the robotic approach demonstrated the lowest rates of overall and major complications (Clavien-Dindo grade ≥ 3).

Our institutional experience confirms what is increasingly evident in high-volume hepatobiliary centers: advanced/expert surgical difficulty alone is no longer a contraindication for a minimally invasive approach. In a high-volume center with standardized protocols and experienced surgical teams, advanced/expert resections can be safely and effectively performed using minimally invasive techniques. The IWATE-based stratification ensured comparability between groups and further revealed that the comparative benefits of minimally invasive surgery are not uniform across all levels of technical difficulty. In low/intermediate resections, both LLR and RLR achieved favorable outcomes compared to OLR, with LLR demonstrating particular efficiency in operative time. In advanced/expert resections, however, the advantages of the robotic platform became most evident, with significantly lower complication rates and shorter hospital stays compared to both LLR and OLR. These findings highlight that surgical difficulty modifies the comparative benefits of each approach.

Beyond the well-described technical advantages of the robotic platform, such as improved instrument articulation, stable three-dimensional visualization, and superior ergonomics, the successful and safe implementation of RLR at our center was undoubtedly facilitated by our long-standing experience in LLR. RLR was introduced at our center in 2021, after performing approximately 300 laparoscopic liver resections since 2018. This established expertise provided a solid foundation for adopting the robotic approach and likely contributed to the low conversion rates and favorable outcomes observed in the RLR group. Operative time, however, was significantly longer for RLR compared to OLR and LLR, consistent with previous reports [7]. This difference is mainly attributable to setup and docking procedures, instrument changes, and the learning curve inherent to robotic systems [7]. Given the relatively limited number of RLR cases in our series (*n* = 101), further reductions in operative time are likely with increasing experience and institutional learning. In addition, it should be acknowledged that the later introduction of RLR may have influenced outcomes through case selection. To account for this potential bias, we included the year of surgery as a covariate in our multivariable analyses, which confirmed the robustness of our findings. Importantly, 75% of robotic resections in our cohort were classified as advanced/expert cases, indicating that the robotic approach at our institution was not confined to technically easier procedures.

To further account for potential confounding, we performed multivariable regression analyses with different model specifications. Across all models, both LLR and RLR remained independently associated with reduced major complication rates and shorter postoperative stay compared to OLR, confirming the robustness of our results. Moreover, interaction term analyses demonstrated that surgical difficulty significantly modified the effect of surgical approach: while differences between minimally invasive and open resections were evident across all IWATE levels, the benefits of RLR were most pronounced in advanced/expert cases.

In this context, it is noteworthy that LLR in the low/intermediate subgroup was not only faster than RLR but also significantly faster than OLR. This observation highlights the procedural efficiency of laparoscopy in less complex resections and supports its use as the standard approach in patients with low/intermediate resections. The shorter operative duration may be partly explained by smaller incisions, reduced closure time, and less extensive mobilization.

Several previous studies have reported an increased rate of bile leakage after RLR, which was mainly attributed to the limited availability of dedicated energy devices and transection tools for robotic liver surgery compared to open or laparoscopic approaches [10, 24, 25]. However, this finding could not be confirmed in our study. We observed no significant differences in the incidence of postoperative bile leakage between RLR and LLR, neither in low/intermediate nor in advanced/expert resections. This is most likely a result of the standardized transection technique applied at our center. The so-called “scissor hepatectomy” technique is based on a combination of blunt and sharp dissection with monopolar robotic scissors in combination with bipolar energy [23]. Gentle traction with bipolar or fenestrated forceps opens the transection plane, while the monopolar scissors are used both with slightly opened tips for lateral spreading movements and with closed tips for forward dissection. Small structures (< 3–5 mm) are coagulated and divided with monopolar or bipolar energy, whereas larger vessels and ducts are dissected, clipped, or ligated individually. Minor oozing can be effectively managed with brief applications of bipolar or monopolar energy. By allowing meticulous exposure and targeted control of intrahepatic structures, this technique ensures precise hemostasis and bile duct sealing without the need for additional energy devices. The technique has been described in greater detail in a dedicated publication from our group [23].

Notably, in our study, the rate of patients with previous liver resections was significantly higher in the OLR group compared to the minimally invasive groups. This difference may, at least in part, explain the higher postoperative complication rate observed after OLR, as repeat hepatectomy is generally considered a risk factor for increased surgical complexity and postoperative morbidity [26]. However, we have previously analyzed this subgroup in detail in a separate study from our center and found no relevant differences in postoperative complication rates between open repeat hepatectomy and minimally invasive repeat hepatectomy, suggesting that a history of previous liver surgery alone does not necessarily result in worse perioperative outcomes when appropriate surgical expertise is available [13]. Additionally, our multivariable regression models that specifically adjusted for repeat hepatectomy likewise confirmed the robustness of our results, indicating that the favorable outcomes after minimally invasive liver resection were not driven by differences in the proportion of repeat procedures.

Another important observation in our cohort is the predominance of parenchyma-sparing resections. Non-anatomic resections and mono- and bisegmentectomies accounted for more than 75% of all procedures. This surgical strategy aims to preserve as much functional liver parenchyma as possible, an essential factor for both postoperative recovery and long-term outcomes [27, 28]. The associated technical complexity is consciously accepted, as the long-term benefits for patients clearly outweigh the operative challenges. Furthermore, parenchyma-sparing resections may facilitate future repeat hepatectomies, particularly in cases of tumor recurrence or metachronous liver lesions.

Despite the strengths of our study, including the large cohort size, the standardized surgical techniques, and the use of the IWATE score for objective risk stratification, several limitations must be acknowledged. First, this was a single-center analysis with a potential selection bias due to its retrospective nature. To minimize bias, we applied well-defined inclusion and exclusion criteria and standardized definitions for all variables. Although the IWATE score provided an objective stratification of surgical difficulty, the final decision regarding the surgical approach was ultimately left to the discretion of the operating surgeon, which may have introduced selection bias. However, it should be emphasized that the study period started in 2018, while robotic liver surgery was only introduced at our center in 2021. Therefore, the potential for selection bias regarding the choice of surgical approach is inherently limited for the earlier years of the study, as robotic-assisted liver resection was not yet available. This temporal aspect of the study design may have contributed to a more balanced comparison of surgical approaches.

Second, the study was conducted at a high-volume hepatobiliary center with extensive expertise in minimally invasive liver surgery, which may limit the generalizability of our findings to lower-volume institutions or centers with limited experience in laparoscopic and robotic liver surgery.

Finally, this study focused on short-term perioperative outcomes. Long-term oncological outcomes, including disease-free and overall survival, were not within the scope of this analysis and require further prospective investigation.

Taken together, our data argue for a rethinking of current surgical paradigms. In many regions, procedures such as cholecystectomy and fundoplication are performed almost exclusively via minimally invasive techniques. Based on the evidence presented here, it is reasonable to advocate for MILS as the standard of care for liver resections, with open approaches reserved for exceptional circumstances.

In conclusion, our study confirms that minimally invasive liver surgery, including RLR, is associated with significant perioperative benefits across all levels of technical difficulty. Seven years after the publication of the Southampton Guidelines, our findings support a renewed call for the broader adoption of MILS as a standard approach. In low/intermediate resections, LLR demonstrated particular efficiency and should be considered the default approach. In contrast, in advanced/expert resections, RLR provided distinct advantages in terms of safety and postoperative outcomes, highlighting the clinical relevance of robotic platforms in technically demanding cases. Thus, the IWATE score not only stratifies operative risk but also serves as a meaningful modifier of the comparative strengths of different surgical approaches, which may guide future decision-making and the allocation of minimally invasive techniques in liver surgery.

## Supplementary Information

Below is the link to the electronic supplementary material.

Supplementary file1 (DOCX 45 KB)

## Data Availability

The data that support the findings of this study are available on request from the corresponding author.
