# Supplementary material for: Robotic-assisted versus laparoscopic versus open liver resection: comparison of postoperative outcomes according to the IWATE difficulty score
Source: Surg Endosc. 2025 Sep 17;39(12):8209–19. doi: 10.1007/s00464-025-12231-0 (PMC12708814; doi:10.1007/s00464-025-12231-0)
Supplement: Supplementary file 1 — Supplementary file1 (DOCX 45 KB) [file 464_2025_12231_MOESM1_ESM.docx]

**Supplementary Table 1: Multivariable logistic regression for major morbidity (Clavien–Dindo ≥III)**

**Model A: Fully adjusted model for all baseline covariates and IWATE score**

| **Variables** | **OR** | **95%CI** | ***p*-value** | |
| --- | --- | --- | --- | --- |
| **Approach** |  |  |  |  |
| LLR vs. OLR | 0.24 | 0.10 – 0.55 | ***<0.001*** | |
| RLR vs. OLR | 0.24 | 0.06 – 1.00 | ***0.032*** | |
| RLR vs. LLR | 0.98 | 0.29 – 3.25 | 0.982 | |
| **Covariates** |  |  |  |  |
| **Age, years** | 1.02 | 0.99 – 1.05 | 0.231 | |
| **BMI, kg/m^2^** | 1.03 | 0.97 – 1.09 | 0.412 | |
| **Gender** (Female vs. Male) | 0.69 | 0.36 – 1.31 | 0.255 | |
| **ASA Score** | 1.26 | 0.77 – 2.04 | 0.354 | |
| **Cardiovascular comorbidities** (Yes vs. No) | 1.50 | 0.71 – 3.19 | 0.288 | |
| **Diabetes mellitus** (Yes vs. No) | 1.03 | 0.52 – 2.02 | 0.943 | |
| **Liver cirrhosis** (Child B vs. Child A) | 1.58 | 0.69 – 3.61 | 0.276 | |
| **Previous treatment** |  |  |  | |
| Previous abdominal surgery (Yes vs. No) | 0.86 | 0.38 – 1.95 | 0.731 | |
| Previous liver resection (Yes vs. No) | 2.55 | 1.30 – 5.01 | ***0.007*** | |
| Previous locoregional therapy (Yes vs. No) | 0.39 | 0.07 – 2.15 | 0.280 | |
| Previous systemic treatment (Yes vs. No) | 1.03 | 0.43 – 2.50 | 0.945 | |
| **Diagnosis** (malignant vs. benign) | 1.15 | 0.72 – 1.86 | 0.559 | |
| **Tumor Size** | 1.05 | 0.68 – 2.03 | 0.266 | |
| **Year of resection** | 1.08 | 0.87 – 1.33 | 0.488 | |
| **Multiple resections** (Yes vs. No) | 0.98 | 0.51 – 1.89 | 0.948 | |
| **IWATE score** | 1.28 | 1.14 – 1.44 | ***<0.001*** | |

**Model B: Parsimonious model adjusted for repeat hepatectomy and multiple resection sites**

| **Variables** | **OR** | **95%CI** | ***p*-value** | |
| --- | --- | --- | --- | --- |
| **Approach** |  |  |  |  |
| LLR vs. OLR | 0.45 | 0.26 – 0.78 | ***0.004*** | |
| RLR vs. OLR | 0.38 | 0.13 – 1.11 | ***0.049*** | |
| RLR vs. LLR | 0.83 | 1.28 – 3.73 | 0.73 | |
| **Covariates** |  |  |  |  |
| Multiple resections (Yes vs. No) | 1.50 | 0.92 – 2.45 | 0.10 | |
| Previous liver resection (Yes vs. No) | 1.89 | 1.01 – 3.27 | 0.02 | |

**Model C: Parsimonious model adjusted for age, sex, BMI and ASA Score**

| **Variables** | **OR** | **95%CI** | ***p*-value** | |
| --- | --- | --- | --- | --- |
| **Approach** |  |  |  |  |
| LLR vs. OLR | 0.42 | 0.23 – 0.75 | ***0.003*** | |
| RLR vs. OLR | 0.40 | 0.24 – 1.01 | ***0.048*** | |
| RLR vs. LLR | 0.94 | 0.36 – 2.49 | 0.98 | |
| **Covariates** |  |  |  |  |
| Age, years | 1.02 | 1.00 – 1.05 | ***0.048*** | |
| BMI, kg/m^2^ | 1.03 | 0.95 – 1.05 | 0.906 | |
| Gender (Female vs. Male) | 0.55 | 0.36 – 0.93 | 0.029 | |
| ASA Score | 1.39 | 0.77 – 2.04 | 0.110 | |

*OR* odds ratio; *CI* confidence interval; *LLR* laparoscopic liver resection; *RLR* robotic liver resection; *OLR* open liver resection; *LOS* length of stay; *ASA* American Society of Anesthesiologists; *BMI*  body mass index

Binary variables are coded as *yes vs. no*; continuous variables are modeled per one-unit increase. For categorical variables, the reference category is indicated in the table.

**Supplementary Table 2: Multivariable linear regression for postoperative length of stay**

**Model A: Fully adjusted model for all baseline covariates and IWATE score**

| **Variables** | **Estimate** | **95%CI** | ***p*-value** | |
| --- | --- | --- | --- | --- |
| **Approach** |  |  |  |  |
| LLR vs. OLR | -11.80 | -16.19 – -7.41 | ***<0.001*** | |
| RLR vs. OLR | -13.94 | -21.17 – -6.33 | ***<0.001*** | |
| RLR vs. LLR | -2.27 | -7.10 – 2.54 | 0.351 | |
| **Covariates** |  |  |  |  |
| **Age, years** | +0.13 | -0.01 – 0.21 | 0.066 | |
| **BMI, kg/m^2^** | -0.07 | -0.31 – 0.07 | 0.642 | |
| **Gender** (Female vs. Male) | -3.25 | -6.56 – 0.07 | 0.071 | |
| **ASA Score** | 1.40 | -1.46 – 4.26 | 0.339 | |
| **Cardiovascular comorbidities** (Yes vs. No) | -2.90 | -7.04 – 1.26 | 0.173 | |
| **Diabetes mellitus** (Yes vs. No) | -0.65 | -4.91 – 3.61 | 0.767 | |
| **Liver cirrhosis** (Yes vs. No) | -0.03 | -5.09 – 5.09 | 0.992 | |
| **Previous treatment** |  |  |  | |
| Previous abdominal surgery (Yes vs. No) | 2.13 | 0.46 – 3.95 | 0.651 | |
| Previous liver resection (Yes vs. No) | -0.70 | -4.10 – 2.70 | 0.745 | |
| Previous locoregional therapy (Yes vs. No) | -1.68 | -10.11 – 7.15 | 0.701 | |
| Previous systemic treatment (Yes vs. No) | -3.07 | -7.99 – 1.85 | 0.222 | |
| **Diagnosis** (malignant vs. benign) | 0.26 | -2.05 – 2.57 | 0.822 | |
| **Tumor Size** | 1.04 | 0.51 – 1.96 | 0.167 | |
| **Year of resection** | 0.12 | -0.09 – 0.11 | 0.841 | |
| **Multiple resections** (Yes vs. No) | 0.83 | -0.84 – 2.01 | 0.421 | |
| **IWATE score** | 0.96 | 0.30 – 1.61 | 0.002 | |

**Model B: Parsimonious model adjusted for repeat hepatectomy and multiple resection sites**

| **Variables** | **Estimate** | **95%CI** | ***p*-value** | |
| --- | --- | --- | --- | --- |
| **Approach** |  |  |  |  |
| LLR vs. OLR | -7.78 | -0.11.10 – -4.46 | ***<0.001*** | |
| RLR vs. OLR | -9.14 | -13.76 – -4.52 | ***<0.001*** | |
| RLR vs. LLR | -1.43 | -4.76 – 1.91 | 0.401 | |
| **Covariates** |  |  |  |  |
| Multiple resections (Yes vs. No) | 0.03 | -1.35 – 1.41 | 0.961 | |
| Previous liver resection (Yes vs. No) | 0.45 | -1.85 – 2.76 | 0.697 | |

**Model C: Parsimonious model adjusted for age, sex, BMI and ASA Score**

| **Variables** | **Estimate** | **95%CI** | ***p*-value** | |
| --- | --- | --- | --- | --- |
| **Approach** |  |  |  |  |
| LLR vs. OLR | -8.03 | -11.59 – -4.47 | ***<0.001*** | |
| RLR vs. OLR | -8.06 | -13.16 – -2.96 | ***0.002*** | |
| RLR vs. LLR | 0.03 | -4.46 – -4.38 | 0.98 | |
| **Covariates** |  |  |  |  |
| Age, years | 0.09 | -0.01 – -0.21 | 0.08 | |
| BMI, kg/m^2^ | -0.14 | -0.40 – 0.11 | 0.26 | |
| Gender (Female vs. Male) | -3.62 | -6.61 – -2.38 | ***0.02*** | |
| ASA Score | 0.62 | -1.71 – 2.97 | 0.59 | |

*OR* odds ratio; *CI* confidence interval; *LLR* laparoscopic liver resection; *RLR* robotic liver resection; *OLR* open liver resection; *LOS* length of stay; *ASA* American Society of Anesthesiologists; *BMI*  body mass index

Binary variables are coded as *yes vs. no*; continuous variables are modeled per one-unit increase. For categorical variables, the reference category is indicated in the table.
